# Immune infiltration and a ferroptosis-associated gene signature for predicting the prognosis of patients with endometrial cancer

**DOI:** 10.18632/aging.203190

**Published:** 2021-06-24

**Authors:** Yin Weijiao, Liao Fuchun, Chen Mengjie, Qin Xiaoqing, Lai Hao, Lin Yuan, Yao Desheng

**Affiliations:** 1 Department of Gynecologic Oncology, Guangxi Medical University Cancer Hospital, Nanning, Guangxi Zhuang Autonomous Region 530021, PR China; 2 Henan Key Laboratory of Cancer Epigenetics, Cancer Hospital, The First Affiliated Hospital, College of Clinical Medicine, Medical College of Henan University of Science and Technology, Luoyang, PR China; 3 Department of Gastrointestinal Surgery, Guangxi Medical University Cancer Hospital, Nanning, Guangxi Zhuang Autonomous Region 530021, PR China

**Keywords:** ferroptosis, endometrial cancer, prognosis

## Abstract

Ferroptosis, a form of programmed cell death induced by excess iron-dependent lipid peroxidation product accumulation, plays a critical role in cancer. However, there are few reports about ferroptosis in endometrial cancer (EC). This article explores the relationship between ferroptosis-related gene (FRG) expression and prognosis in EC patients. One hundred thirty-five FRGs were obtained by mining the literature, retrieving GeneCards and analyzing 552 malignant uterine corpus endometrial carcinoma (UCEC) samples, which were randomly assigned to training and testing groups (1:1 ratio), and 23 normal samples from The Cancer Genome Atlas (TCGA). We established a signature using eight screened FRGs (*MDM2, GPX4, PRKAA2, PRNP, SLC11A2, ATP5MC3, PHKG2* and *ACO1*) related to overall survival using LASSO regression analysis. The samples were divided into low- and high-risk subgroups according to the median risk score. Kaplan-Meier survival curves showed that the low-risk group had better OS. ROC curves showed that this signature performed well in predicting OS (1-, 2-, 3-, and 5-year AUCs of 0.676, 0.775, 0.797, and 0.826, respectively). We systematically analyzed the immune infiltrating profile in UCEC samples from TCGA. Overall, our study identified a novel prognostic signature of 8 FRGs that can potentially predict the prognosis of EC.

## INTRODUCTION

Endometrial cancer (EC) is the leading gynecologic tumor in developed countries and remains the second most frequently occurring tumor in developing countries. With the decline in birth rate and the growing epidemic of obesity, its incidence rate has increased significantly. It was reported that there were an estimated 382,000 new cases and 89,900 deaths worldwide in 2018 [1]. Although surgical treatment provides early-stage EC patients with a good prognosis, the 5-year OS of relapsed or metastatic EC patients is decreased dramatically [2]. Therefore, careful prognostic evaluation is urgently needed.

Since iron has a unique role and function in the female reproductive system, it is not surprising that iron disorders have been noted in many gynecological diseases [3]. It was reported that iron-mediated cell death (ferroptosis) was closely related to several endometrial diseases, such as endometriosis [3], repeated implantation failure [4], and endometrial hyperplasia [5], and it can be used as a therapeutic target for these diseases [5–7]. Ferroptosis is a novel form of programmed cell death induced by the excess accumulation of iron-dependent lipid peroxidation products [8]. Studies have revealed that ferroptosis is related to the growth and development of EC [9, 10] and various other cancers, such as pancreatic cancer [11], hepatocellular carcinoma [12], gastric cancer [13, 14], colorectal cancer [15, 16], breast cancer [17, 18], lung cancer [19], ovarian cancer [20], clear cell renal cell carcinoma [21, 22], and head and neck cancer [23]. However, the role of ferroptosis in EC remains unclear; thus, it is imperative to explore the relationship between EC and ferroptosis.

To explore the relationship between ferroptosis-related genes (FRGs) and the prognostic value of ferroptosis in EC, we collected 135 FRGs, downloaded 552 UCEC samples from The Cancer Genome Atlas (TCGA) and constructed a prognostic signature containing eight FRGs. The results showed that FRGs may play a critical role in EC.

## RESULTS

### Identification of candidate FRGs

**Figure 1:**
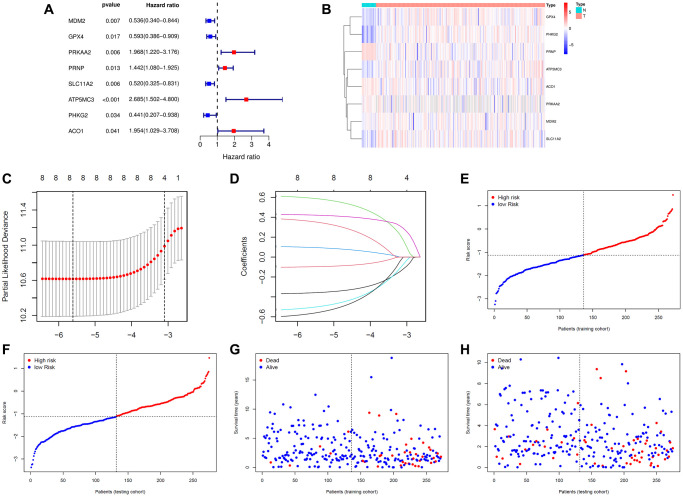
**Construction of the signature.** (**A**) The prognostic analyses for eight ferroptosis-related genes in the training cohort of endometrial cancer using a univariate Cox regression model. Hazard ratio >1 represented risk factors for survival and hazard ratio <1 represented protective factors for survival. (**B**) Heatmap of eight ferroptosis-related genes in 23 normal samples and 272 endometrial cancer samples. (**C**) Optimal parameter (λ) selected in the LASSO Cox regression model based on the minimum criteria. (**D**) The LASSO coefficient profiles of the eight ferroptosis-related genes signature. (**E**) The distribution and median value of the risk scores in the training cohort. (**F**) The distribution and median value of the risk scores in the training cohort. (**G**) Survival statuses of endometrial cancer patients in the training cohort. (**H**) Survival statuses of endometrial cancer patients in the testing cohort.

We obtained 87 differentially expressed ferroptosis-related genes (DE-FRGs) (false discovery rate (FDR) < 0.05) through the Wilcoxon test and the “limma” R package between 23 normal samples and 276 training samples. Then, we extracted 12 prognostic FRGs through univariate Cox analysis implemented by the “survival” R package (*P* < 0.05) (Supplementary Figure 1). Intersecting the 87 DE-FRGs and the 12 prognostic FRGs resulted in eight prognostic FRGs, namely, *MDM2*, *GPX4*, *PRKAA2*, *PRNP*, *SLC11A2*, *ATP5MC3*, *PHKG2* and *ACO1*. From the results of univariate Cox regression analysis, we found that *MDM2*, *GPX4*, *SLC11A2* and *PHKG2* are favorable genes; that is, those with high expression levels of these genes had a good prognosis. *PRKAA2*, *PRNP*, *ATP5MC3* and *ACO1* were unfavorable genes (Figure 1A). From the heatmap in Figure 1B and the mean expression of the eight FRGs in Table 1, we could see that the eight FRGs were abnormally expressed in EC samples compared with normal samples. The expression levels of *GPX4*, *ATP5MC3, SLC11A2,* *PHKG2* and *MDM2* were upregulated in the EC group, while the expression levels of *PRNP*, *ACO1* and *PRKAA2* were higher in the normal group.

**Table 1:** Mean expression and calculated difference value of eight FRGs.

**Gene ID**	**ConMean**	**EC.Mean**	**logFC**	***P* Value**	**FDR**	**Coef**
MDM2	5.209091	7.138429	0.454575	0.024106	0.033182	–0.34216
GPX4	77.67361	197.0067	1.342749	3.29E-10	2.14E-09	–0.08952
PRKAA2	2.082538	1.557735	–0.41889	0.000291	0.000576	0.55497
PRNP	82.51514	18.32231	–2.17106	4.29E-14	1.25E-12	0.08230
SLC11A2	6.368706	12.21955	0.940119	1.06E-05	2.48E-05	–0.46253
ATP5MC3	15.5362	25.16703	0.695901	1.40E-07	4.97E-07	0.41109
PHKG2	2.96983	7.257239	1.28904	1.79E-12	2.09E-11	–0.50883
ACO1	7.499537	5.685589	–0.39949	0.000159	0.000332	0.30930

Abbreviations: ConMean: Mean gene expression in normal samples; FDR: false discovery rate; EC.Mean: Mean gene expression in endometrial cancer samples; Coef: Coefficients in prognostic models.

### Construction and validation of a prognostic FRG signature

**Figure 2:**
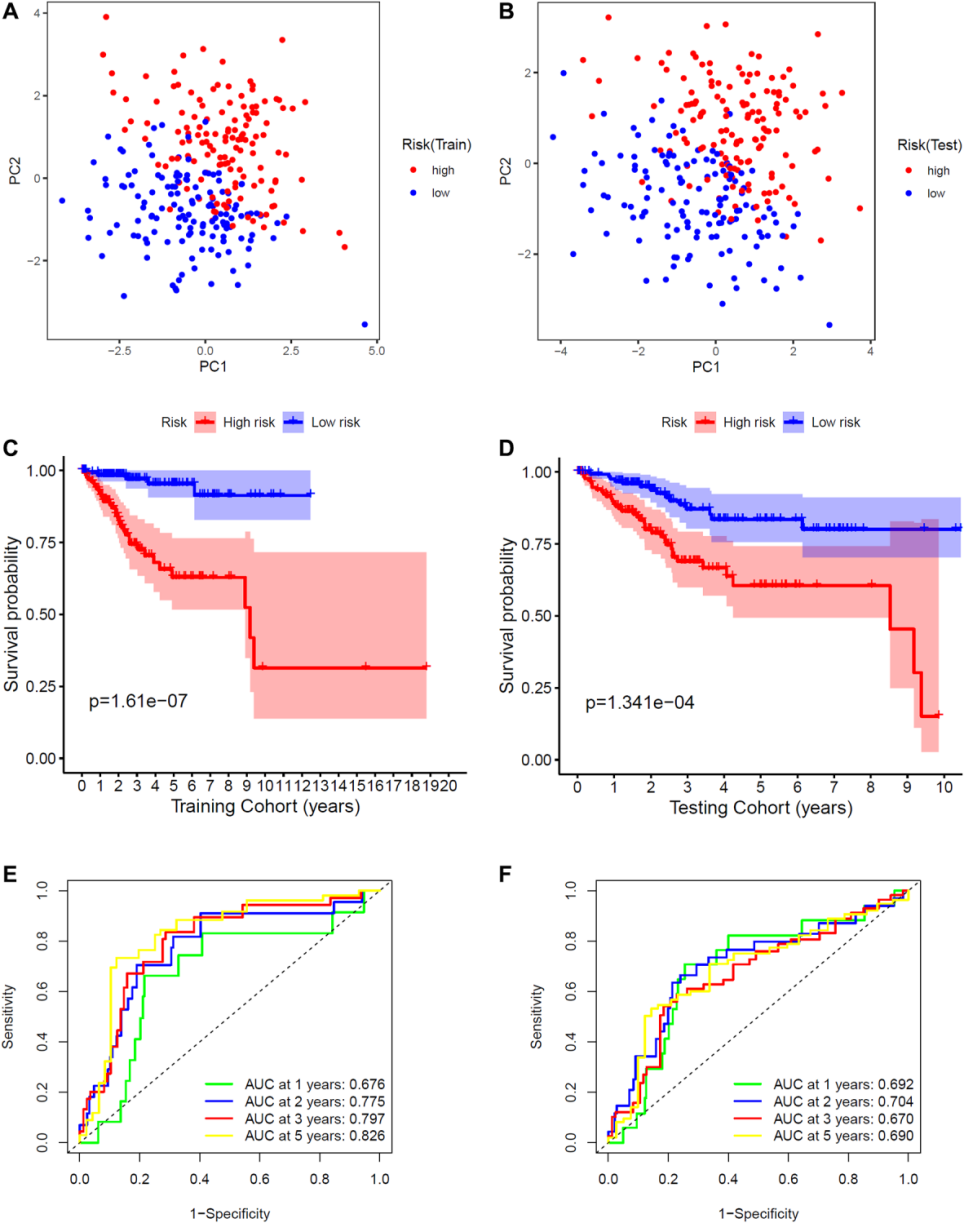
**Validation of the signature.** (**A**) PCA plot in the training cohort. (**B**) PCA plot in the training and testing cohorts. (**C**) K-M survival curve of endometrial cancer patients in the training group. (**D**) K-M survival curve of endometrial cancer patients in the testing group. (**E**) Time-dependent ROC curve of endometrial cancer patients in the training group. (**F**) Time-dependent ROC curve of endometrial cancer patients in the testing group.

Then, these eight FRGs were input into the LASSO regression model for feature selection. Under penalizing conditions (alpha = 1), 8 FRG scores with nonzero coefficients were selected to formulate the risk score: Risk score = (–0.34216 × MDM2 expression) + (–0.08952 × GPX4 expression) + (0.55497 × PRKAA2 expression) + (0.08230 x PRNP expression) + (–0.46253 × SLC11A2 expression) + (0.41109 × ATP5MC3 expression) + (–0.50883 × PHKG2 expression) + (0.30930 × ACO1 expression) (Figure 1C, 1D). According to the median risk score of the training group, the samples were divided into low- and high-risk groups (Figure 1E–1H). By principal component analysis (PCA), we also demonstrated that EC samples in different risk groups were distributed in two directions as a whole (Figure 2A, 2B). The Kaplan-Meier survival curves of the training group showed that the predicted survival time of the low-risk group was obviously longer than that of the high-risk group, *P* < 0.001 (Figure 2C, 2D). Time-dependent receiver operating characteristic (ROC) curves of the EC samples showed that the 1-year area under the curve (AUC) was 0.676, the 2-year AUC was 0.775, the 3-year AUC was 0.797, and the 5-year AUC was 0.826 in the training group, and the 1-year AUC was 0.692, the 2-year AUC was 0.704, the 3-year AUC was 0.670, and the 5-year AUC was 0.690 in the testing group, which indicated that the performance of the 8-FRG signature was very stable (Figure 2E, 2F).

**Figure 3:**
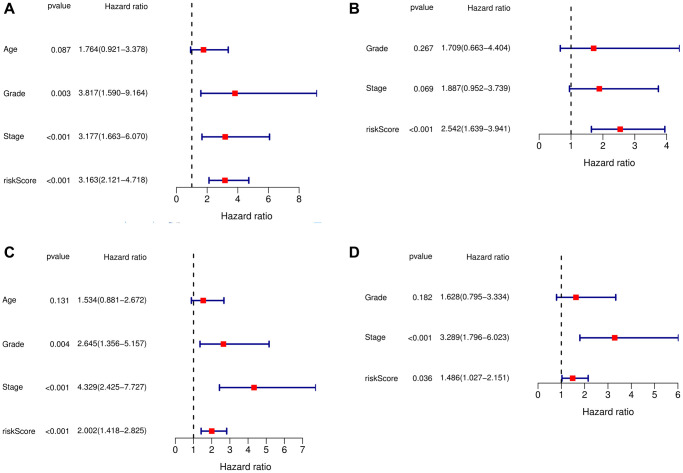
**Independent prognostic analysis of risk scores and clinical parameters.** (**A**) Univariate Cox regression analysis in the training cohort. (**B**) The multivariate Cox regression analysis in the training cohort. (**C**) The univariate Cox regression analysis in the testing cohort. (**D**) The multivariate Cox regression analysis in the testing cohort.

The above analysis showed that this signature performed well. Then, we wanted to determine whether the signature was an independent prognostic factor, so we performed univariate and multivariate Cox regression analyses, which showed that the signature was indeed an independent prognostic factor (Figure 3A–3D).

### Functional annotation

**Figure 4:**
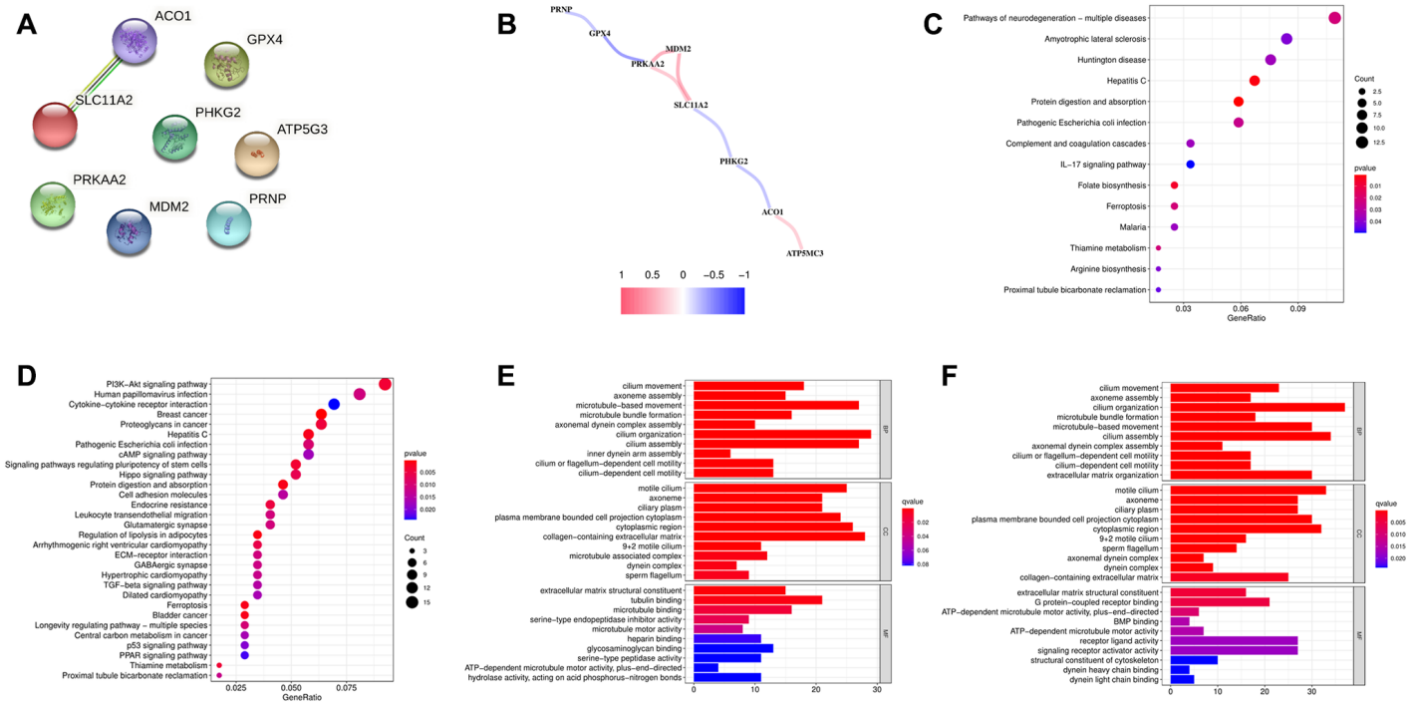
(**A**) PPI of eight ferroptosis-related genes (FRGs). (**B**) Correlation network of the eight FRGs. (**C**) KEGG analysis of the differentially expressed genes (DEGs) between the low- and high-risk groups in the training cohort. (**D**) KEGG analysis of the DEGs between the low- and high-risk groups in the testing cohort. (**E**) GO analysis of the DEGs between the low- and high-risk groups in the training cohort. (**F**) GO analysis of the DEGs between the low- and high-risk groups in the testing cohort.

To observe the expression specificity of these eight FRGs in different tissues of the human body, we searched the Human Protein Atlas (HPA) database. We found that *PHKG2* and *GPX4* were specifically highly expressed in germ cells; *ATP5MC3* was specifically highly expressed in distal tubular cells, proximal tubular cells, Hofbauer cells, extravillous trophoblasts, and cytotrophoblasts; *PRNP* was highly expressed in basal keratinocytes; *ACO1* was highly expressed in hepatocytes and proximal tubular cells; *PRKAA2* was highly expressed in cardiomyocytes and rod photoreceptor cells; and *MDM2* and *SLC11A2* had low cell-type specificity (Supplementary Figure 2A–2J). Then, we explored the relationships among the eight FRGs through Search Tool for the Retrieval of Interacting Genes/Proteins (STRING) and Pearson correlation analysis. The STRING results showed that *SLC11A2* was related to *ACO1*, while the others were independent of each other (Figure 4A). The correlation network of these 8 FRGs showed that there was a positive coexpression correlation among *PRKAA2*, *MDM2* and *SLC11A2*. *ATP5MC3* and *ACO1* had a similar positive correlation. *PRNP* and *GPX4*, *PRKAA2* and *GPX4*, *PHKG2* and *SLC11A2*, and *PHKG2* and *ACO1* showed negative coexpression correlations (Figure 4B).

Kyoto Encyclopedia of Genes and Genomes (KEGG) analysis of these eight FRGs showed that they were mainly related to ferroptosis (Supplementary Figure 3A). Gene Ontology (GO) analysis of the biological processes of these eight FRGs showed that they were mainly related to metal ions. Their molecular functions were mainly involved in proton transmembrane transporter activity and monovalent inorganic cation transmembrane transporter activity (Supplementary Figure 3B). KEGG analysis of the differentially expressed genes (DEGs) between the low- and high-risk groups showed that they were mainly enriched in cancer-associated pathways, such as the PI3K-Akt signaling pathway [24], human papillomavirus infection [25], breast cancer, proteoglycans in cancer, protein digestion and absorption, and hepatitis C [26] (Figure 4C, 4D). GO analysis of biological processes and cellular components showed that the DEGs were mainly related to the cilium. Molecular function analysis showed that the DEGs were mainly related to tubulin binding (Figure 4E, 4F).

### Immune annotation

**Figure 5:**
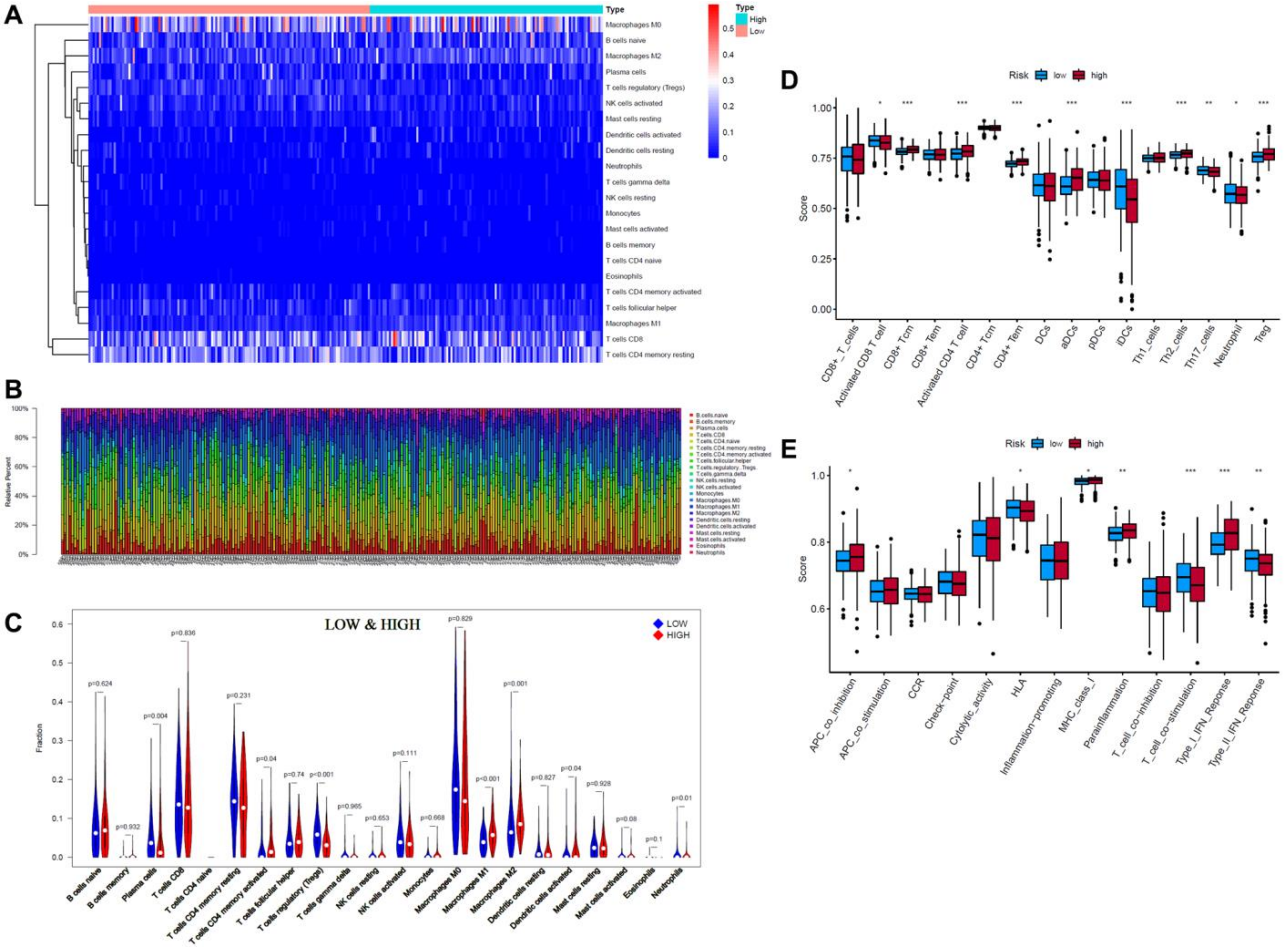
**Tumor-infiltrating immune cells (TIICs) analysis of 243 endometrial cancer (EC) patients (133 low-risk patients and 110 high-risk patients) (CIBERSORT: *P* < 0.05**). (**A**, **B**) Composition of 22 TIICs. (**C**) Wilcoxon test analysis of 22 TIICs between low- and high-risk EC patients. (**D**, **E**) Single-sample gene set enrichment analysis (ssGSEA) of specific immune cells and immune functions (^*^p < 0.05; ^**^p < 0.01; ^***^p < 0.001).

Since ferroptosis is closely related to immunity, we explored the differences in immune infiltration between the high- and low-risk groups. CIBERSORT is a classic method for characterizing the composition of 22 immune cells from gene expression profiles in complex tissues [24], so we used it to analyze the composition of immune cells. Through CIBERSORT algorithm analysis, we obtained 243 EC patients (133 low-risk patients and 110 high-risk patients) whose CIBERSORT results showed *P* < 0.05. The results are presented in the form of a heatmap and bar plot (Figure 5A, 5B). As seen from the figure, the composition of tumor-infiltrating immune cells (TIICs) in the high- and low-risk groups remained basically the same, mainly composed of M0 macrophages, CD8 T cells and resting memory CD4 T cells, with significant differences in some immune cells, such as M2 macrophages. The relatively high expression of CD8 T cells may be the reason why endometrial carcinoma has a better prognosis than highly malignant tumors. It can also be seen that local immunity is not strongly suppressed in these EC patients. Wilcoxon test analysis showed that plasma cells and regulatory T cells (Tregs) showed higher infiltration in the low-risk group than in the high-risk group. Activated memory CD4 T cells, activated dendritic cells (aDCs), M1 macrophages and M2 macrophages showed higher infiltration in the high-risk group (Figure 5C).

Considering the high proportion of CD8 T cells in the high- and low-risk groups, the particularity of activated memory CD4 T cells and the poor display of neutrophils and dendritic cells (DCs) in the violin plot, we further conducted single-sample gene set enrichment analysis (ssGSEA) for these cells and other immune functions (Figure 5D, 5E). The ssGSEA results were consistent with those of CIBERSORT. CD8 T cells, DCs, and plasmacytoid DCs (pDCs) did not differ between the high- and low-risk groups. There was also no difference in effector memory CD8 T cells (CD8+ Tem). However, activated CD8 T cells were more significantly enriched in the low-risk group, while central memory CD8 T cells were more significantly enriched in the high-risk group. Activated CD4 T cells, effector memory CD4 T cells (CD4+ Tem), and aDCs were also more significantly enriched in the high-risk group, while immature dendritic cells (iDCs) and neutrophils were more significantly enriched in the low-risk group. In addition, the ssGSEA results of immune function showed that aDCs, NK cells, APC coinhibition, MHC class I, parainflammation, and type I interferon (IFN) response were more significantly enriched in the high-risk group, while HLA, T cell costimulation, and type II IFN response were more significantly enriched in the low-risk group.

**Figure 6:**
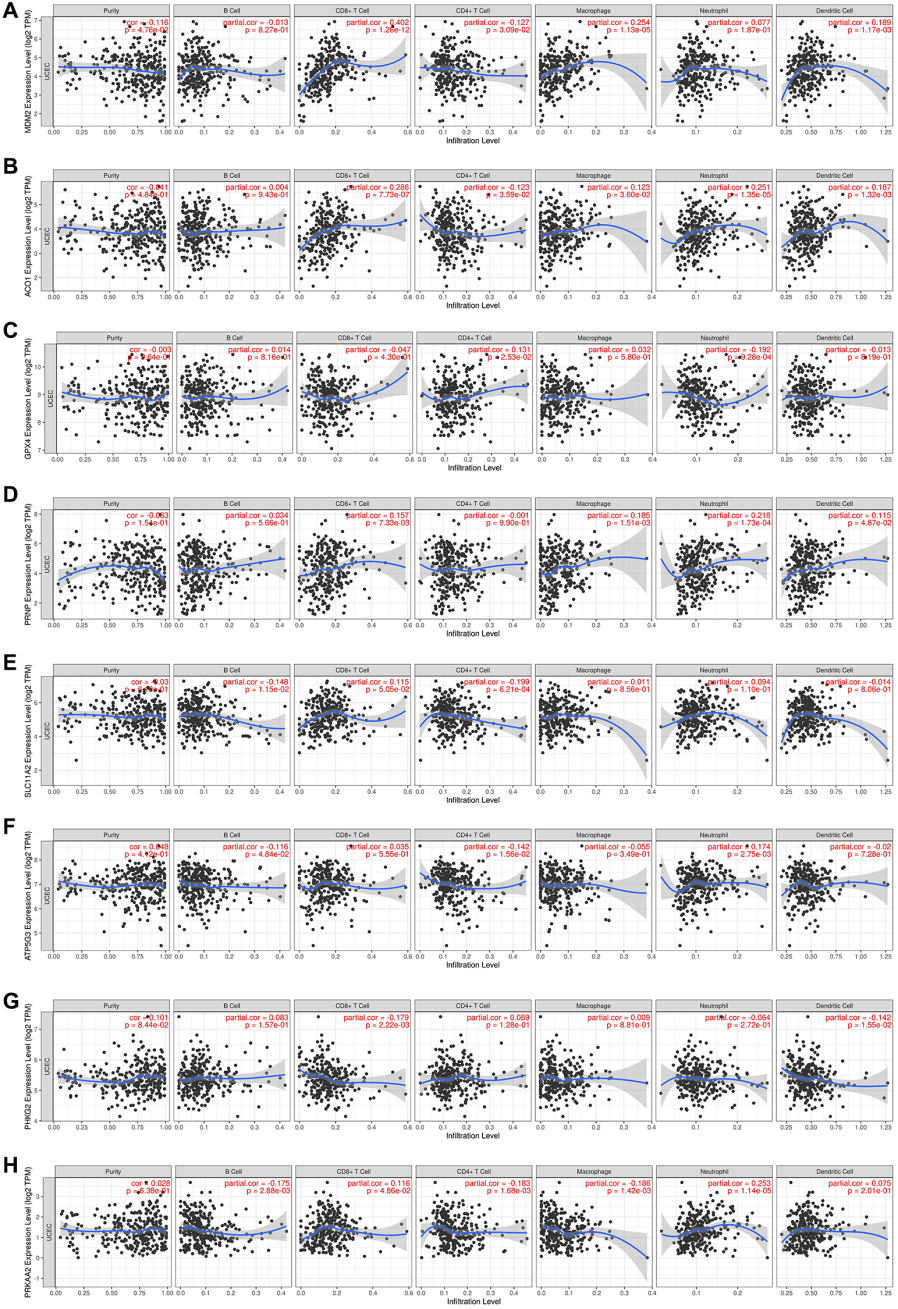
**(A–H)** The TIMER database results of the correlations between the expression of eight ferroptosis-related genes and immune infiltrating cells in endometrial cancer patients, showing the purity-corrected partial Spearman’s rho value and statistical significance.

To better understand the relationship between the eight FRGs and TIICs, we searched the TIMER database (Figure 6A–6H). We could see from the left-most panel, which displays the gene expression levels (log2 TPM) against tumor purity, that *MDM2* expression was higher in the microenvironment in EC (cor = –0.116, *P* = 0.048). *ACO1*, *GPX4*, *PRNP* and *SLC11A2* may be highly expressed in the microenvironment, but the difference was not statistically significant (cor < 0, *P* > 0.05). *ATP5G3*, *PHKG2* and *PRKAA2* may be highly expressed in tumor cells, but the difference was not statistically significant (cor > 0, *P* > 0.05). The results also showed that *ACO1* was positively correlated with macrophages, neutrophils, CD8+ T cells and DCs (*P* < 0.05) and negatively associated with CD4+ T cells (cor = 0.123, *P* = 0.036). *ATP5MC3* was negatively correlated with B cells (*P* = 0.048) and CD4+ T cells and positively correlated with neutrophils (*P* = 0.003). *GPX4* was negatively associated with neutrophils (*P* < 0.001) and positively associated with CD4+ T cells (*P* = 0.025). *MDM2* was significantly positively associated with CD8+ T cells, CD4+ T cells, macrophages and DCs (*P* < 0.05). *PHKG2* was negatively correlated with CD8+ T cells and DCs (cor < 0, *P* < 0.05). *PRKAA2* was significantly negatively correlated with B cells, CD4+ T cells, and macrophages and positively correlated with CD8+ T cells and neutrophils (*P* < 0.05). *PRNP* was significantly positively associated with CD8+ T cells, macrophages, neutrophils and DCs (*P* < 0.05). *SLC11A2* was negatively correlated with B cells and CD4+ T cells.

### Genetic variation

**Figure 7:**
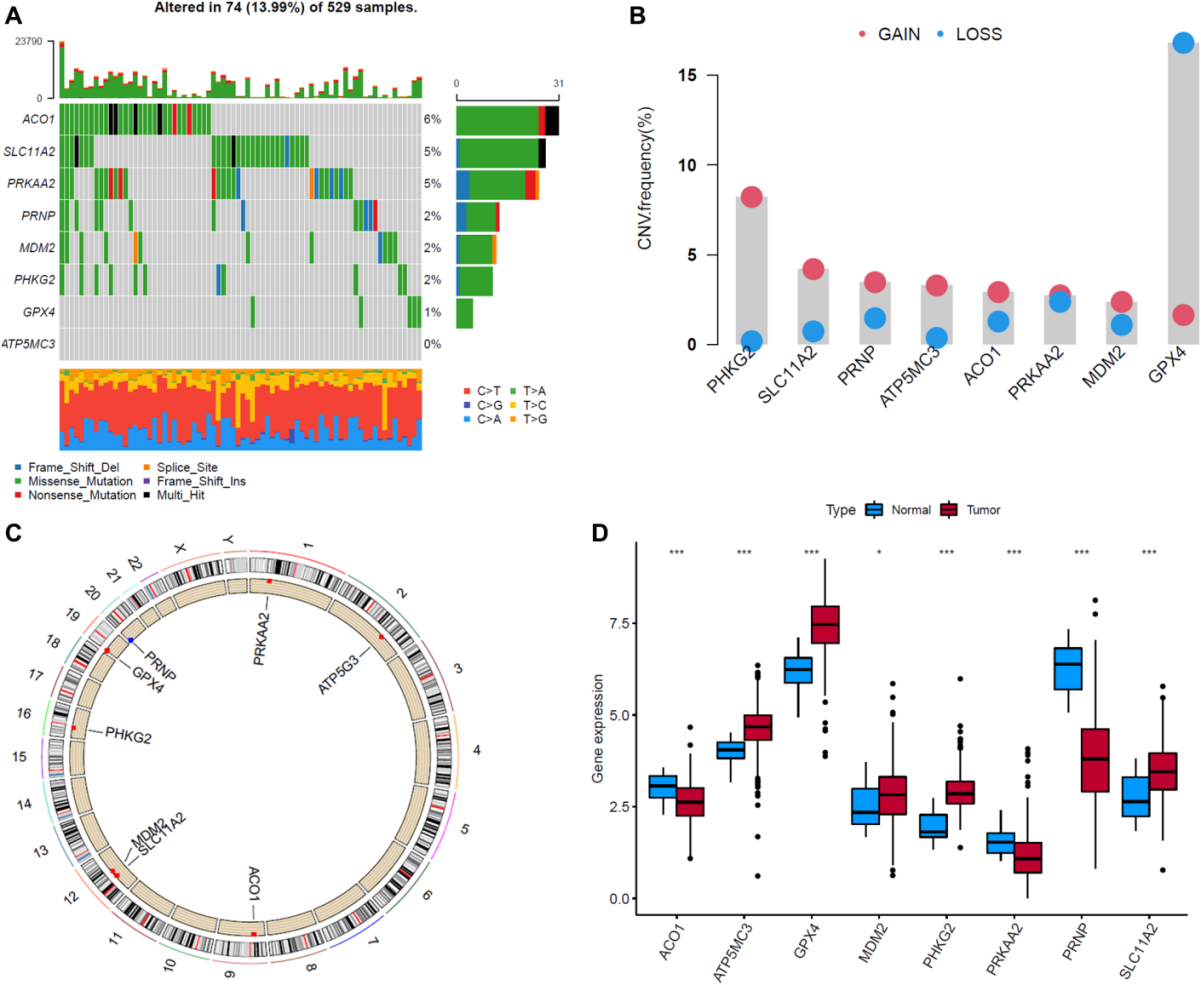
**Landscape of genetic and expression variation of eight ferroptosis-related genes (FRGs) in endometrial cancer (EC) samples.** (**A**) The alteration frequency of 8 FRGs in 529 EC samples. Each column represented individual patients. The upper bar plot showed TMB. The number on the right indicated the alteration frequency in each regulator. The right bar plot showed the proportion of each variant type. The stacked bar plot below showed fraction of conversions in each sample. (**B**) The CNV variation frequency of FRGs in EC samples. The height of the column represented the alteration frequency. The deletion frequency, blue dot; The amplification frequency, red dot. (**C**) The location of CNV alteration of FRGs on 23 chromosomes. (**D**) The expression of 8 FRGs between normal tissues and EC tissues. Tumor, red; Normal, blue. The upper and lower ends of the boxes represented interquartile range of values. The lines in the boxes represented median value, and black dots showed outliers. The asterisks represented the statistical *p* value (^*^P < 0.05; ^**^P < 0.01; ^***^P < 0.001).

We then summarized the incidence of copy number variations (CNV) and somatic mutations of 8 FRGs in EC. Among 529 samples, 74 experienced alterations of 8 FRGs, with frequency 13.99%. The genetic alteration rates of *MDM2*, *GPX4*, *PRKAA2*, *PRNP*, *SLC11A2*, *ATP5MC3*, *PHKG2* and *ACO1* were 2%, 1%, 5%, 2%, 5%, 0%, 2% and 6%, respectively. Most of their genetic alteration type was missense mutation (Figure 7A). The investigation of CNV alteration frequency showed most were focused on the amplification in copy number, while GPX4 had a widespread frequency of CNV deletion (Figure 7B). The location of CNV alteration of FRGs on chromosomes was shown in Figure 7C. To ascertain whether the above genetic alterations influenced the expression of FRGs in EC patients, we investigated the mRNA expression levels of FRGs between normal and EC samples, and found that the alterations of CNV was not the prominent factors resulting in perturbations on the GPX4, ACO1 and PRNP expression. Compared to normal endometrial tissues, genes with amplificated CNV demonstrated markedly lower expression in EC tissues (e.g., ACO1 and PRNP), and vice versa (e.g., GPX4) (Figure 7C, 7D). The alterations of CNV could be the prominent factors resulting in perturbations on the PHKG2, SLC11A2 and ATP5MC3 expression. They exhibited higher expression in EC tissues, companied with amplificated CNV. The above analyses indicate a high degree of heterogeneity in the landscape of genetic and expressional alterations in FRGs between normal and EC samples.

## DISCUSSION

EC is a heterogeneous disease. Although surgery can provide favorable survival in early-stage EC patients, the treatment measures and prognosis of advanced and metastatic EC remain poor. Since iron plays a critical role in the female reproductive system, iron-mediated cell death (ferroptosis) aroused our attention. A previous study found that cell death pathways, such as necroptosis and ferroptosis, are present in low-grade, early-stage endometrioid EC [10]. Interestingly, these proteomic research [10] results are consistent with our research results, namely, macrophage and CD8 T cell infiltration was significantly increased in EC tissues. Meanwhile, there was heterogeneity in the immune response between tumors (Figure 5A, 5B) [25]. In our study, TIICs in EC were analyzed by CIBERSORT, ssGSEA and the TIMER database. As seen in Figure 5C, 5D patients in the low-risk group had a better immune response to kill tumor cells, namely, more plasma cells and activated CD8+ T cells, and more enrichment in immune function of T cell costimulation and type II IFN response. T-cell costimulation is a hierarchical process with elements of mutual interdependence [26]. It is often essential for the development of an effective immune response. Type II IFN is mainly produced by activated T cells and NK cells. Although the proportion of Tregs in the low-risk group was significantly higher than that in the high-risk group, according to the calculation results of CIBERSORT, the enrichment score of Tregs in ssGSEA was significantly lower than that in the high-risk group. Combined with the greater enrichment of APC coinhibition of immune function, antitumor immunity was suppressed in the high-risk group. Tregs are an important subset of CD4+ T cells. Their function is to maintain immune homeostasis. Loss of function of Tregs can lead to autoimmunity, while excessive activity of Tregs can promote tumorigenesis [27]. Higher Tregs may be the result of higher parainflammation and a type I IFN response. Parainflammation is a type of inflammation between homeostasis and chronic inflammation. Numerous studies have shown that parainflammation is widely present in tumors and correlates with poor prognosis [28]. The function of pDCs is mainly to secrete type I IFN. Although the enrichment score of pDCs showed no difference between the high- and low-risk groups, the enrichment of type I IFN-responsive genes was significantly higher in the high-risk group. Type I IFN is mainly activated by viruses and responds to viral infection [29, 30].

T-helper 17 (Th17) cells can mobilize, recruit and activate neutrophils. In our results, Th17 cells were significantly enriched in the low-risk group. Th17 cells have also been suggested to be an independent prognostic factor for the survival of squamous cervical cancer and are significantly associated with improved disease-specific survival [31]. Kryczek et al. reported that decreased tumor ascites Th17 cells are a significant predictor of increased risk for reduced survival in ovarian cancer [32]. Th2 cells secrete IL-4 and IL-13, which can promote the differentiation of macrophages into M2 macrophages. Macrophages are cells that differentiate from mononuclear cells in the blood when they pass through the blood vessels. It is a plastic and pluripotent cell population, showing obvious functional differences under the influence of different microenvironments. In this article, M2 macrophages had a much higher proportion than M1 macrophages. M2 macrophages were more common in the high-risk group than in the low-risk group, which predicted a poorer prognosis. M2 is the pro-tumor subtype of macrophages. Jin et al. reported that TBBPA (a novel organic contaminant widely detected in human samples)-driven M2 macrophage polarization is responsible for EC deterioration [33]. M2 macrophage–conditioned medium treated with selective estrogen receptor alpha (ERα) agonists induced epithelial-to-mesenchymal transformation (EMT) in EC cells [34]. Although the high-risk group was enriched with more APCs (aDCs, activated CD4 T cells, MCH I class, etc.), its antigen-presenting function was significantly suppressed (higher enrichment of APC coinhibition).

In summary, our results showed that the low-risk group had a greater immune ability to kill tumor cells, while the immune response of the high-risk group was more strongly suppressed.

Glutathione peroxidase 4 (GPX4) is a key regulator of ferroptosis that catalyzes the reduction of hydrogen peroxide, organic hydroperoxides, and lipid hydroperoxides, thereby protecting cells against oxidative damage. It can also repair lipid peroxides as an antioxidant enzyme and regulate cytokine signaling [35, 36]. This is consistent with our findings: *GPX4* is a favorable prognostic factor in EC patients. *PRKAA2* encodes a catalytic subunit of AMP-activated protein kinase (AMPK), an important energy-sensing enzyme that monitors cellular energy status. HPA is a favorable prognostic marker for renal cancer and an unfavorable prognostic marker for liver cancer and EC, which is consistent with our results. In addition, high expression of *PRKAA2* may predict poor prognosis in head and neck squamous cell carcinoma [37] and colorectal cancer (CRC) [38]. MDM2 is a p53 regulator and encodes a nuclear-localized E3 ubiquitin ligase that keeps the activity of p53 low under normal conditions by targeting *p53* for degradation via the 26S proteasome [39]. In response to various oncogenic stresses, the p19ARF protein product of the INK4a locus binds to and disables the E3 ligase activity of MDM2; thus, hyperproliferative signals activate p53[40]. The MDM2 promoter SNP55 (rs2870820) T-allele was also associated with a reduced risk of endometrial cancer before 50 years of age [41]. However, the single-cell specificity of *MDM2* was low (Supplementary Figure 2D). The TIMER results showed that *MDM2* expression was higher in the microenvironment in EC and strongly positively associated with CD8+ T cells (cor = 0.402, *P* = 1.26e-12) (Figure 6A). The RNA blood cell type of *MDM2* also showed that it was commonly expressed in all immune cells (Supplementary Figure 2J). Therefore, high levels of *MDM2* and CD8+ T cells in the tumor microenvironment may be the reason why EC patients have a better OS. Zhou et al. reported that mice with MDM2-deficient T cells showed accelerated tumor progression and decreased survival and function of tumor-infiltrating CD8+ T cells [42]. Therefore, it is interesting to conduct further research on MDM2-CD8+ T cell-p53 in EC. SLC11A2 is the only known transmembrane iron transporter involved in cellular iron uptake, acting as a proton-dependent iron importer of Fe^2+ ^[43]. Its relationship with cancer has not been reported. Our study shows that the expression of *SLC11A2* is upregulated in EC, and it is positively coexpressed with *MDM2*. PHKG2 is a phosphorylase kinase. Methylation of *PHKG2* may be associated with mutation of the *BRAF/RAS* oncogene in papillary thyroid cancer [44]. It is differentially expressed in paired normal tissue samples and EC samples [45] and could be inhibited by alectinib, which can strongly inhibit RET activity [46]. However, studies about its relationship with cancer are rare. The prion protein (PRNP) gene encodes a membrane glycosylphosphatidylinositol-anchored glycoprotein (prion protein, PrP^C^) that tends to aggregate into rod-like structures. It is involved in the main aspects of cancer biology: proliferation, metastasis, and drug resistance [47]. PRNP is associated with the prognosis of many cancers, such as gastric cancer [48], CRC [49] and pancreatic cancer [50]. Therefore, it is not surprising that it is a poor prognostic factor for EC. Reports on the correlation of ATP5MC3 (also known as *P3* and *ATP5G3*) with cancer are lacking. However, according to data from the HPA database, it is a potentially favorable prognostic marker in renal cancer and CRC and a potentially unfavorable prognostic marker in head and neck cancer. In our reports, ATP5MC3 was an unfavorable prognostic factor for EC. Aconitase 1 (ACO1) encodes a bifunctional, cytosolic protein that functions as an essential enzyme in the TCA cycle and interacts with mRNA to control intracellular iron levels. When the iron content is high, this protein binds to 4Fe-4S and acts as an aconitase, catalyzing the conversion of citrate to isocitrate. When cellular iron levels are low, this protein binds to the iron response element, thereby inhibiting the translation of ferritin mRNA and the degradation of rapidly degrading transferrin receptor mRNA. It may be associated with tumor development and progression [51, 52].

This study also has limitations. First, the data were analyzed from the TCGA database. However, the sample size was large enough that we could perform random grouping to analyze and verify the data. Second, due to the scarcity of studies about ferroptosis in EC, the prognostic value and mechanisms of ferroptosis still need further validation in more studies.

In conclusion, we constructed a prognostic FRG signature in EC for the first time. We also validated that it can be a prognostic indicator independent of other clinical factors in EC. Since studies on the relationship between ferroptosis and EC are still rare, this study can also provide ideas and directions for ferroptosis-related studies in EC, which may be favorable for novel therapeutic methods.

## MATERIALS AND METHODS

### Datasets and FRGs

Gene expression quantification RNA-Seq (HTSeq-FPKM) of transcriptome profiling and clinical data of uterine corpus endometrial carcinoma (UCEC) were downloaded from The Cancer Genome Atlas (TCGA) website (https://portal.gdc.cancer.gov), including 552 malignant tumor samples and 23 normal samples. A total of 552 malignant tumor samples were randomly assigned to two groups in a 1:1 ratio. Then, malignant tumor samples with incomplete key clinical information (age, grade, stage, survival time, survival status) were omitted, leaving 272 samples in the training group and 273 samples in the test group (Supplementary Table 1). A total of 103 ferroptosis-related genes (FRGs) were retrieved from GeneCards (https://www.genecards.org/), and 60 FRGs were retrieved from previous literature [20]. Intersecting the two sets of genes resulted in 135 FRGs (Supplementary Table 2). Finally, the expression of the eight genes in single cells was also analyzed in the Human Protein Atlas (HPA) database (https://www.proteinatlas.org).

### Construction of the FRG signature

First, we screened the differentially expressed FRGs (DE-FRGs) between normal samples and EC patients in the training set by using the “limma” R package with *P* < 0.05 and false discovery rate (FDR) < 0.05. Then, we identified twelve potential prognostic FRGs in the training set through univariate Cox analysis of OS by using the coxph function in the “survival” R package with *P* < 0.05 (Supplementary Figure 4). Intersecting the two sets of genes resulted in eight FRGs (Supplementary Figure 1). Second, we put these eight prognostic FRGs into the least absolute shrinkage and selection operator (LASSO) regression model. The LASSO analysis with cross-validation was conducted by the “glmnet” R package [53, 54].

### Verification of the FRG signature

The patients were classified into low- and high-risk subgroups based on the median risk score of the training group. We performed survival analysis to compare the OS between the high- and low-risk groups and showed results via Kaplan-Meier curves. Multivariate Cox analysis, receiver operating characteristic (ROC) analysis, and principal component analysis (PCA) were also performed to test the specificity and sensitivity of the survival prediction.

### Functional annotation analysis

After verifying that this 8-FRG signature performed well, we obtained the differentially expressed genes (DEGs) between the low- and high-risk groups of EC patients in the training and testing groups by the “limma” R package and performed Gene Ontology (GO) and Kyoto Encyclopedia of Genes and Genomes (KEGG) enrichment analyses of the DEGs by using the “clusterProfiler” R package.

### Immune cells and immune-related functional annotation

We analyzed the proportion of 22 tumor-infiltrating immune cells (TIICs) in all malignant tumor samples via the CIBERSORT algorithm [55, 56] and compared the difference in infiltrating scores of 13 immune-related pathways and 16 immune cells between the low- and high-risk groups by using single-sample gene set enrichment analysis (ssGSEA) in the "GSVA" R package [57]. Finally, we also explored the correlation of the eight prognostic FRGs with immune cells in EC patients through the TIMER database [58, 59]. In addition, simple nucleotide variation dataset (workflow type: VarScan2 Variant Aggregation and Masking) of UCEC were downloaded from TCGA and analyzed by the “maftools” R package. Copy number (gene-level) of TCGA-UCEC were downloaded from UCSC Xena (http://xena.ucsc.edu/) and analyzed by the “RCircos” R package.

### Statistical analysis

In this study, all statistical analyses were performed using R software (version 3.6.3). Continuous variables were compared using the Wilcoxon test. Survival analyses were conducted using the Kaplan–Meier method with the log-rank test by the “survival” R package. Feature selection was conducted with univariate and multivariate Cox regression. Time-dependent ROC curve analysis and LASSO Cox regression analysis with cross-validation were performed using R packages. Unless otherwise stated, statistical significance was defined at *p* values < 0.05.

## Supplementary Materials

Supplementary Figures

Supplementary Tables
