# Supplementary material for: Immune infiltration and a ferroptosis-associated gene signature for predicting the prognosis of patients with endometrial cancer
Source: Aging (Albany NY). 2021 Jun 24;13(12):16713–32. doi: 10.18632/aging.203190 (PMC8266342; doi:10.18632/aging.203190)
Supplement: Supplementary Figures [file aging-13-203190-s001.pdf]

SUPPLEMENTARY FIGURES

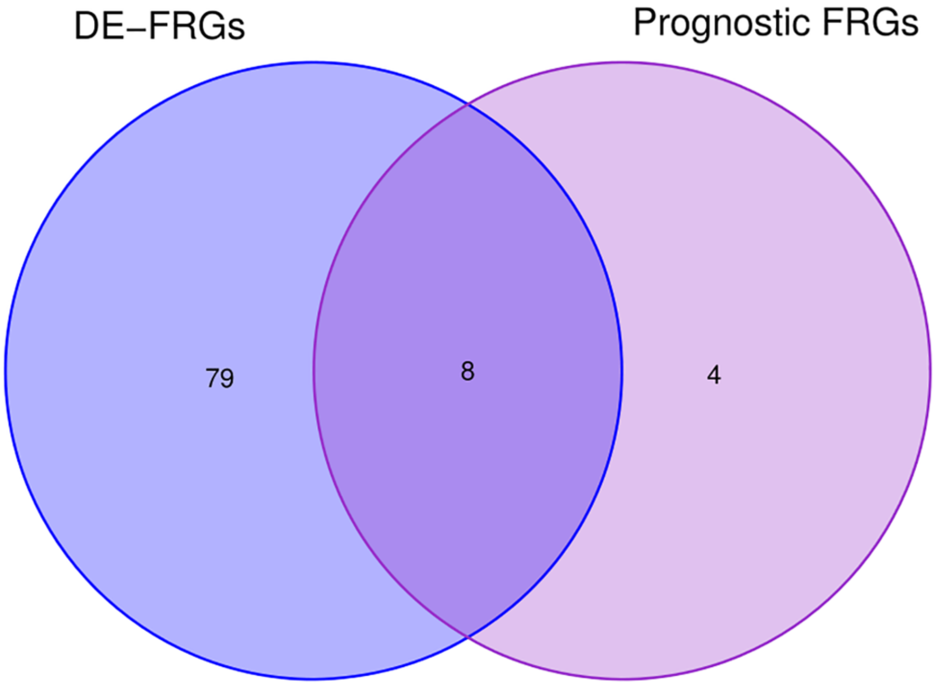

Supplementary Figure 1. Venn diagram of 87 differentially expressed ferroptosis-related genes (DE-FRGs) between normal samples and EC patient samples and 12 prognostic FRGs.

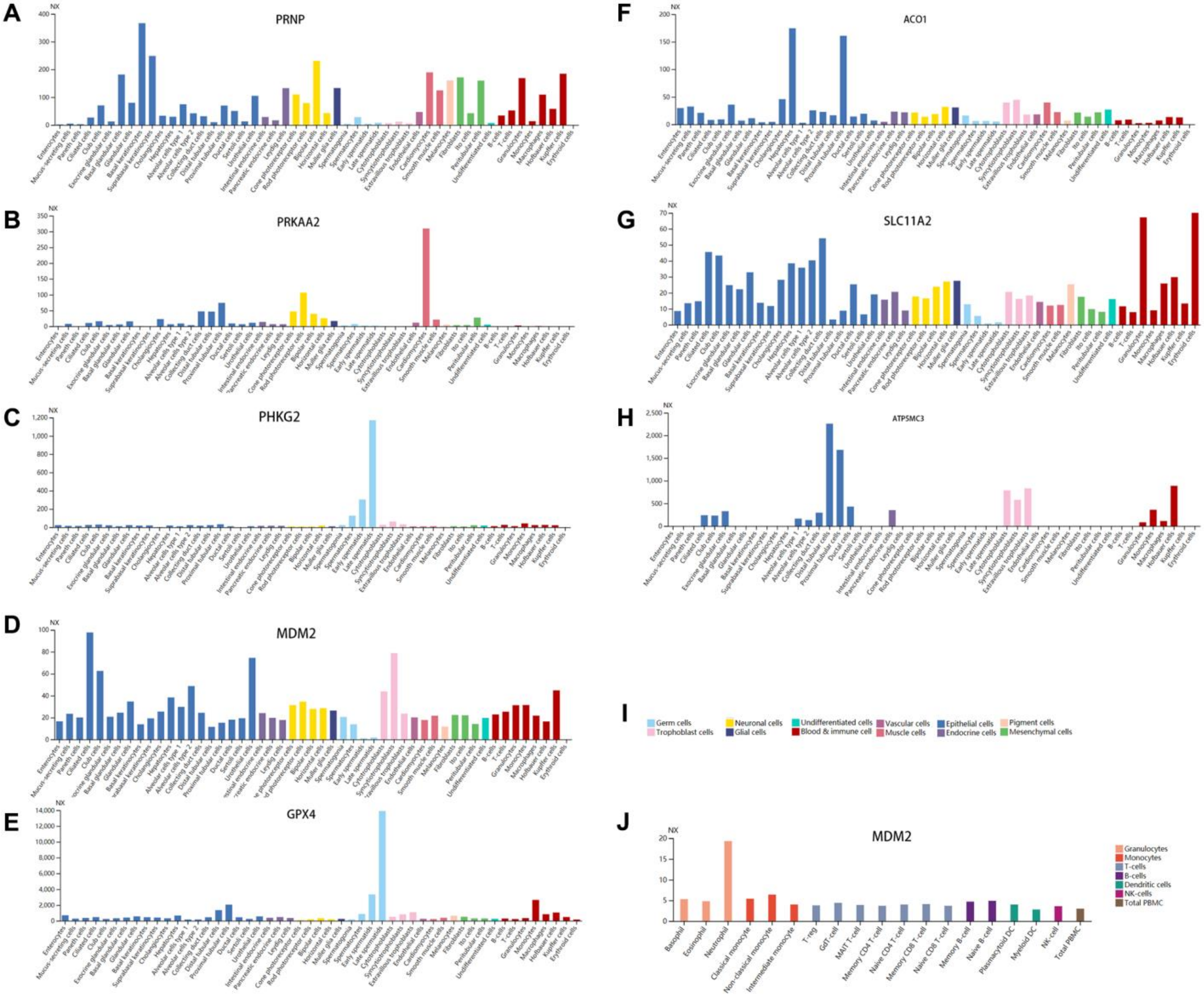

Supplementary Figure 2. (**A**–**I**) RNA single-cell analysis of 8 FRGs in normal human tissue cells. (**J**) MDM2 RNA single-cell analysis in blood cells.

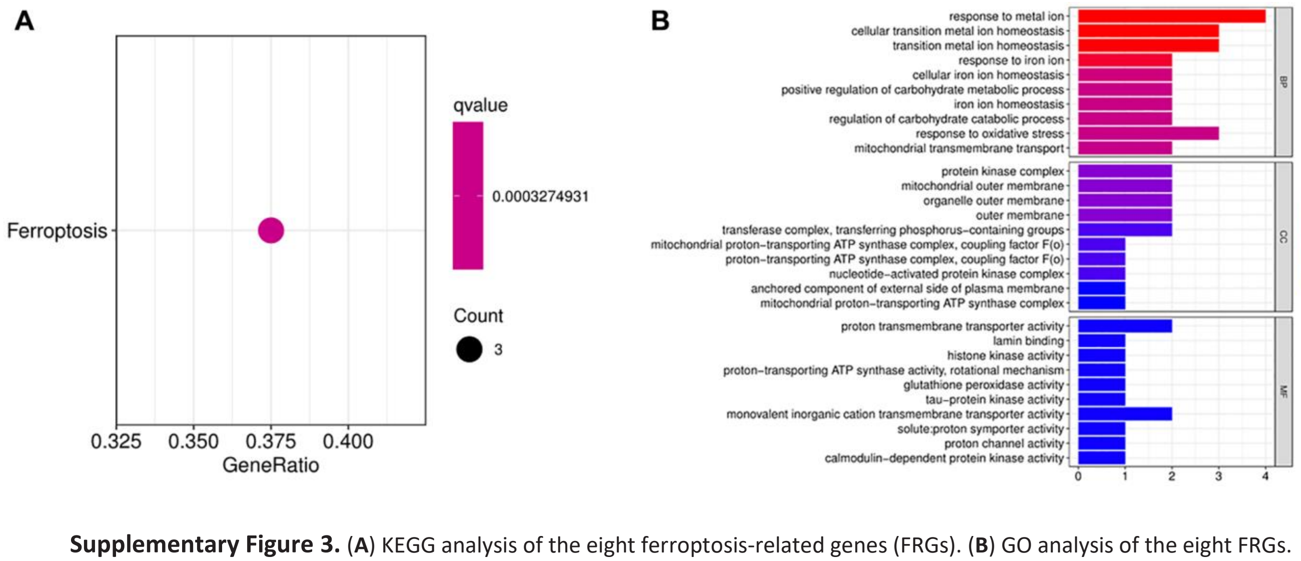

Supplementary Figure 3. (**A**) KEGG analysis of the eight ferroptosis-related genes (FRGs). (**B**) GO analysis of the eight FRGs.

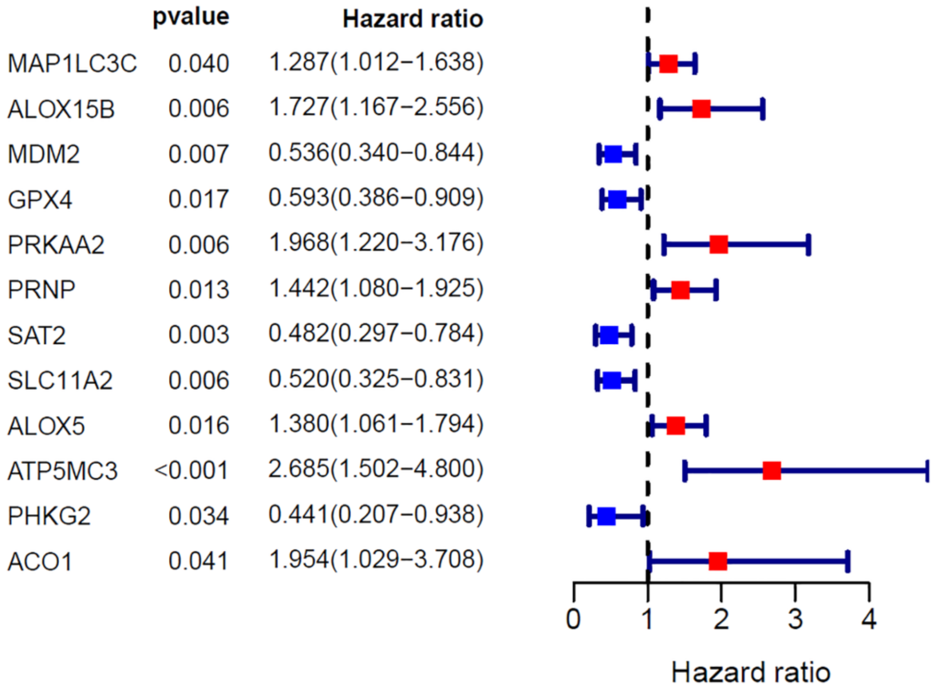

Supplementary Figure 4. Results of the univariate Cox analysis of the twelve prognostic ferroptosis-related genes in EC patients.
