# Supplementary material for: Immune infiltration and a ferroptosis-associated gene signature for predicting the prognosis of patients with endometrial cancer
Source: Aging (Albany NY). 2021 Jun 24;13(12):16713–32. doi: 10.18632/aging.203190 (PMC8266342; doi:10.18632/aging.203190)
Supplement: Supplementary Tables [file aging-13-203190-s002.pdf]

SUPPLEMENTARY TABLES

Supplementary Table 1. Clinical characteristics of the patients included in the study.

Characteristics		Training Cohort	Validation Cohort
AGE	≤65	161	161
	>65	111	112
GRADE	<3	107	100
	≥3	165	173
STAGE	≤2	194	185
	>2	78	88
DIABETES	NO	140	133
	YES	47	54
	NA	85	86
HYPERTENSION	NO	91	84
	YES	109	116
	NA	72	73
RACE	Asian	9	11
	black	53	56
	white	184	187
	other	8	4
	NA	18	15
Total sample num.		272	273

Supplementary Table 2. List of ferroptosis-related genes.

	Total	Elements
GCards.txt text_mining.txt	28	MT1G CD44 GPX4 STEAP3 HSPB1 GCLM TP53 FTH1 HMOX1 ALOX12 SLC7A11 TFRC GOT1 CARS1 PEBP1 ACSL3 NCOA4 ALOX15 CISD1 GSS GCLC ACSL4 AIFM2 SAT1 NFS1 LPCAT3 FANCD2 NFE2L2
GCards.txt	75	FH HSPA5 BECN1 MUC1 EPAS1 SLC39A8 PRKAA2 VDAC1 VDAC3 G3BP1 SLC40A1 MAP1LC3A ACSL5 OTUB1 SLC11A2 TF FTMT CDKN2A PCBP1 MAP1LC3B2 MIR9-1 RIPK1 MIR7-1 MYC ACSL1 HELLS LAMP2 ALOX15B NF2 PROM2 CASP8 SESN2 PRDX6 NGB CYBB MAP1LC3B ACSL6 SLC39A14 ELAVL1 ATG5 SLC3A2 MAP1LC3C MIR137 EGLN1 TIGAR MAP3K5 CP ATF4 ARNTL ATG7 MAPK1 HILPDA ANO6 PRNP SOCS1 LINC00472 CA9 PRKAA1 YAP1 RB1 CFTR MDM2 AURKA VDAC2 SAT2 MIF BAP1 NEDD4 PCBP2 ITGA6 GUCY1A1 HMGB1 FTL LINC00336 PRC1
text_mining.txt	32	HMGCR AKR1C3 CRYAB NOX1 G6PD ZEB1 AKR1C1 KEAP1 ALOX5 EMC2 CS PGD PHKG2 ACACA HSBP1 SLC1A5 AKR1C2 RPL8 FDFT1 FADS2 PTGS2 SQLE GLS2 CBS ABCC1 CHAC1 DPP4 NQO1 IREB2 ACO1 ATP5MC3 ACSF2
